# A novel variant c.1185_1196dup (p.(Gly396_Ser399dup)) in the SLC26A4 gene associated with recessive hearing loss

**DOI:** 10.18699/vjgb-26-82

**Published:** 2026-09

**Authors:** M.V. Zytsar, V.Yu. Danilchenko, A.A. Bondar, K.E. Orishchenko, O.L. Posukh

**Affiliations:** Institute of Cytology and Genetics of the Siberian Branch of the Russian Academy of Sciences, Novosibirsk, Russia Novosibirsk State University, Novosibirsk, Russia; Institute of Cytology and Genetics of the Siberian Branch of the Russian Academy of Sciences, Novosibirsk, Russia Novosibirsk State University, Novosibirsk, Russia; Knorre Institute of Chemical Biology and Fundamental Medicine of the Siberian Branch of the Russian Academy of Sciences, Novosibirsk, Russia; Institute of Cytology and Genetics of the Siberian Branch of the Russian Academy of Sciences, Novosibirsk, Russia Novosibirsk State University, Novosibirsk, Russia; Institute of Cytology and Genetics of the Siberian Branch of the Russian Academy of Sciences, Novosibirsk, Russia Novosibirsk State University, Novosibirsk, Russia

**Keywords:** hearing loss, SLC26A4, pathogenic variants of the SLC26A4 gene, MLPA method, indels, потеря слуха, патогенные варианты гена SLC26A4, метод MLPA, инделы

## Abstract

Pathogenic variants in the SLC26A4 gene (solute carrier family 26, member 4) are a common cause of inherited hearing loss. The SLC26A4 gene encodes the transmembrane protein pendrin, a member of the SLC26 anion transporter family, with predominant expression in the inner ear, thyroid, and kidney tissues. Pathogenic SLC26A4 variants cause nonsyndromic recessive hearing loss (DFNB4) and Pendred syndrome (sensorineural hearing loss and thyroid dysfunction). In many studies analyzing the SLC26A4 gene, along with patients who have two recessive pathogenic SLC26A4 variants (M2 patients) and an accurate molecular genetic diagnosis can be made, a group of patients with only one pathogenic SLC26A4 variant (M1 patients) is often identified, which creates a diagnostic problem. The presence of M1 patients may reflect copy number variations (CNVs, deletions/duplications) in the SLC26A4 gene that are not detected by routine diagnostic methods. The aim of the study is to search for deletions/duplications in the SLC26A4 gene using the MLPA (Multiplex Ligation-dependent Probe Amplification) method in thirteen patients belonging to the indigenous people of the Tyva Republic (Southern Siberia), in whom only one pathogenic SLC26A4 variant (M1 patients) was identified during the study of the etiology of hereditary hearing loss. As a result of MLPA analysis and molecular cloning in DNA samples of three M1 patients from two related Tuvinian families, a novel variant was revealed – tandem duplication of twelve nucleotides (c.1185_1196dup) in exon 10 of the SLC26A4 gene. This variant is an in-frame insertion resulting in the inclusion of four additional amino acid residues Gly-Phe-Phe-Ser (p.(Gly396_Ser399dup)) in a highly conserved region of the pendrin protein. Most predictive programs predict the damaging effect of the c.1185_1196dup variant on the structure and function of the pendrin protein. The pathogenicity of the c.1185_1196dup variant is supported by its segregation with hearing pathology in the pedigree of patients in whom it was found in a compound heterozygous state with pathogenic SLC26A4 variants, as well as its absence in a control sample of Tuvinians and in the world genomic databases. Functional in vitro studies are needed to confirm the potentially deleterious effects of the novel c.1185_1196dup (p.(Gly396_Ser399dup)) variant and its association with hearing pathology.

## Abstract (ru)

Патогенные варианты в гене SLC26A4 (solute carrier family 26, member 4) – частая причина наследственной потери слуха. Ген SLC26A4 кодирует трансмембранный белок пендрин из семейства анионных транспортеров SLC26, с преимущественной экспрессией в тканях внутреннего уха, щитовидной железы и почек.Патогенные SLC26A4-варианты вызывают несиндромальную рецессивно наследуемую потерю слуха (DFNB4) и синдром Пендреда (сенсоневральная тугоухость и дисфункция щитовидной железы). Во многих исследованиях при анализе гена SLC26A4, наряду с пациентами, у которых обнаружено два рецессивных патогенных SLC26A4- варианта (М2-пациенты) и может быть поставлен точный молекулярно-генетический диагноз, часто выявляется группа пациентов только с одним патогенным SLC26A4-вариантом (М1-пациенты), что создает диагностическую проблему. Наличие М1-пациентов может отражать не обнаруженные рутинными методами диагностики вариации числа копий (CNV, делеции/дупликации в гене) SLC26A4. Цель работы – поиск делеций/дупликаций в гене SLC26A4 методом MLPA (Multiplex Ligation-dependent Probe Amplification) у 13 пациентов, относящихся к коренному населению Республики Тыва (Южная Сибирь), у которых при изучении этиологии наследственной потери слуха был найден только один патогенный SLC26A4-вариант (М1-пациенты). В результате MLPA-анализа и молекулярного клонирования в образцах ДНК трех М1-пациентов из двух родственных тувинских семей обнаружен новый вариант – тандемная дупликация 12 нуклеотидов (c.1185_1196dup) в экзоне 10 гена SLC26A4, которая является внутрирамочной инсерцией, приводящей к включению четырех дополнительных аминокислотных остатков Gly-Phe-Phe-Ser (p.(Gly396_Ser399dup)) в высококонсервативном районе белка пендрин. Бóльшая часть предсказательных программ прогнозирует повреждающее воздействие варианта c.1185_1196dup на структуру и функцию белка пендрин. В пользу патогенности свидетельствует его сегрегация с патологией слуха в родословной пациентов, у которых он найден в компаунд-гетерозиготном состоянии с патогенными SLC26A4- вариантами, а также его отсутствие в контрольной выборке тувинцев и мировых базах геномных данных. Для подтверждения потенциально негативных эффектов нового варианта c.1185_1196dup (p.(Gly396_Ser399dup)) и его ассоциации с патологией слуха необходимы функциональные in vitro исследования.

## Introduction

Hereditary hearing loss is a monogenic disorder characterized by exceptionally high genetic heterogeneity. It may occur as an isolated condition or as one of the clinical features of numerous distinct syndromes. According to the Hereditary Hearing Loss Homepage (https://hereditaryhearingloss.org, accessed April 8, 2026), pathogenic variants in over 150 different genes – including those located in mitochondrial DNA – are associated with nonsyndromic (isolated) forms of hearing loss. These forms exhibit diverse inheritance patterns, including autosomal dominant (DFNA loci), autosomal recessive (DFNB loci), and X-linked (DFNX loci). Pathogenic variants in the GJB2 gene (gap junction protein beta-2, 13q12.11, OMIM #121011), which encodes the transmembrane protein connexin 26, are responsible for nonsyndromic autosomal recessive deafness type 1A (DFNB1A, OMIM #220290) and represent one of the most prevalent forms of hearing loss across many populations (del Castillo F., del Castillo I., 2017). Pathogenic variants in the SLC26A4 gene (solute carrier family 26, member 4, 7q22.3, OMIM #605646) constitute the second most common cause of inherited hearing loss, at least in Asian populations (Park et al., 2003; Albert et al., 2006; Yuan et al., 2009; Du et al., 2013; Tsukada et al., 2015; Koohiyan, 2019; Lee et al., 2023).

The SLC26A4 gene encodes protein pendrin, an anion transporter belonging to the SLC26 family. Pendrin is predominantly expressed in the inner ear, thyroid gland, and kidneys, where it mediates the transport of various anions (Everett et al., 1997; Mount, Romero, 2004). In the inner ear, pendrin maintains endolymph homeostasis via Cl–/HCO– 3 anion exchange, whereas in thyroid cells it facilitates I– efflux from follicular cells into the follicular lumen (Everett et al., 1997; Wangemann et al., 2007; Tesolin et al., 2021). Pathogenic variants in SLC26A4 disrupt the structure and function of pendrin and can lead to either nonsyndromic recessive hearing loss (DFNB4, OMIM #600791) or Pendred syndrome (OMIM #274600), which is characterized by sensorineural hearing loss and thyroid dysfunction (goiter). An important clinical feature of SLC26A4-associated hearing loss is an enlarged vestibular aqueduct (EVA, OMIM #603545), along with other malformations of the inner ear; these are typically identified by computed tomography (CT) of the temporal bones.

Clinical manifestations in patients with pathogenic SLC26A4 variants are highly variable. Hearing loss may be congenital or manifest later in life, with severity ranging from moderate impairment to profound deafness and often showing significant progression. Similarly, the extent of inner ear malformations and the onset and severity of thyroid dysfunction in Pendred syndrome vary widely (Wangemann et al., 2007; Griffith, Wangemann, 2011; Mey et al., 2019; Roesch et al., 2021; Honda, Griffith, 2022).

Screening for pathogenic SLC26A4 variants is an important part of molecular genetic diagnostics for hearing impairment, particularly in patients with EVA and other anomalies of the hearing organ. Nevertheless, the contribution of SLC26A4 to the etiology of hearing loss remains incompletely characterized. There is considerable variability across studies in the proportion of patients with biallelic pathogenic SLC26A4 variants (M2 patients) and those with only a single pathogenic variant (M1 patients) (Azaiez et al., 2007; Choi B. et al., 2009; Chattaraj et al., 2013; Pique et al., 2014; Rose et al., 2017; Smits et al., 2022; Tian et al., 2024). Moreover, a substantial number of individuals exhibiting clinical features typical of SLC26A4-related hearing loss do not show any detectable pathogenic variants in this gene (M0 patients). This variability in diagnostic yields can be caused by several factors: heterogeneity in patient cohorts (differing in size and inclusion criteria); potential contributions from other genes or environmental factors (Yang et al., 2007, 2009; Pique et al., 2014; Chattaraj et al., 2017; Chao et al., 2019; Smits et al., 2022); and differences in the methodologies used to analyze the SLC26A4 gene. These range from targeted screening of common pathogenic variants to comprehensive analysis of coding and flanking intronic regions or the entire gene sequence via Sanger sequencing and/or next-generation sequencing (NGS) technologies. Notably, the relatively high proportion of M1 patients reported in many studies may reflect the presence of undetected pathogenic variants or copy number variations (CNVs) in the SLC26A4 gene.

CNVs (deletions or duplications ranging from 50–100 bp to several megabases) are well-established causes of numerous genetic disorders, including hereditary hearing loss (Zhang et al., 2009; Stankiewicz, Lupski, 2010; Shearer et al., 2014; Rentas, Abou Tayoun, 2021; Royer-Bertrand et al., 2021).

Among the methods developed for detecting such deletions and duplications, Multiplex Ligation-dependent Probe Amplification (MLPA) is considered one of the most promising approaches (Schouten et al., 2002; Stuppia et al., 2012). The technique relies on hybridization of two probe segments to the target DNA sequence, followed by ligation and PCR amplification with universal fluorescently labeled primers. Each amplified product has a distinct length, enabling simultaneous detection of multiple fragments by capillary electrophoresis. The signal intensity of each PCR product correlates with the copy number of the corresponding DNA fragment; thus, comparison with reference samples allows identification of copy number alterations in specific genomic regions.

To date, several large deletions affecting one or more exons of the SLC26A4 gene have been identified using various molecular genetic methods, including MLPA (Park et al., 2003; Hu H. et al., 2007; Pera et al., 2008; Anwar et al., 2009; Pique et al., 2014; Pang et al., 2015; Cirello et al., 2018; Lin et al., 2019; Tian et al., 2024). Although most studies report that the frequency of CNVs in SLC26A4-associated hearing loss is low (typically a few percent or less), CNV testing is recommended as part of the diagnostic workflow for patients with unresolved etiologies, particularly among M1 individuals in whom only a single pathogenic SLC26A4 variant has been identified.

In a previous study investigating the etiology of hereditary hearing loss in the indigenous population of the Tyva Republic (Southern Siberia), a notably high proportion of Tuvinian patients (62/220, 28.2 %) was found to carry biallelic pathogenic SLC26A4 variants in either a homozygous or compound heterozygous state (M2 patients), thereby enabling a molecular diagnosis of recessively inherited hearing loss due to SLC26A4 pathogenic variants. Additionally, 13 patients were identified as M1 carriers, harboring only one pathogenic SLC26A4 variant (Danilchenko et al., 2021). The aim of the present study is to investigate deletions and duplications in the SLC26A4 gene using the MLPA method in a group of M1 patients who carry a single pathogenic variant of this gene.

## Materials and methods

**Samples analyzed**

MLPA analysis was carried out on a group of 13 Tuvinian patients (M1 patients) in whom only a single recessive pathogenic variant of the SLC26A4 gene had been identified during the initial molecular screening (Danilchenko et al., 2021). Additionally, a control sample comprising 129 ethnically matched (Tuvinians) healthy individuals without hearing impairment was used to screen for the novel c.1185_1196dup variant.

**Experimental methods**

Multiplex Ligation-dependent Probe Amplification (MLPA). Deletions and duplications in the SLC26A4 gene were investigated using MLPA with the SALSA MLPA Probemix P280‑B4 SLC26A4 kit (MRC-Holland, Netherlands). The kit includes 24 probes: 21 targeting each exon of the SLC26A4 gene and 3 designed to detect point mutations c.707T>C, IVS8+1G>A (c.1001+1G>A), and c.1246A>C. MLPA was performed according to the manufacturer’s protocol. Amplified MLPA products were separated by capillary electrophoresis on a 3130XL Genetic Analyzer (Applied Biosystems, USA). Data analysis was conducted using Coffalyser. Net software (https://www.mrcholland.com/).

Molecular cloning. DNA samples from patients harboring the novel c.1185_1196dup variant (exon 10) of the SLC26A4 gene were used as templates for molecular cloning. Exon 10 was amplified using specific primers (5ʹ-ACC TCTAGAAGATGGTATGGCGT-3ʹ and 5ʹ-GCTAGGATCC GTGCGAGCCTTCCT-3ʹ), which contain XbaI and BamHI restriction endonuclease recognition sites at their 5ʹ-ends, respectively. PCR products and the pUC18 plasmid vector were digested with XbaI and BamHI (New England Biolabs, USA). Subsequently, PCR products were purified using Agencourt Ampure XP magnetic beads (Beckman Coulter, USA), and the pUC18 vector was isolated from a 1 % agarose gel with the QIAEX II Gel Extraction Kit (Qiagen, Netherlands). The purified PCR fragment was ligated into the prepared pUC18 vector using T4 DNA ligase (SibEnzyme, Russia). The ligation mixture was purified on Cleanup S-Cap columns (Eurogen, Russia) and used to transform E. coli XL1-blue cells via electroporation. Recombinant clones carrying the target DNA fragment were selected by PCR colony screening 14–16 h after transformation, using primers 5ʹ-GTGGAATTGTGAGCGG ATAAC-3ʹ and 5ʹ-TCCCAGTCACGACGTTGTAAA-3ʹ. The nucleotide sequence of the insert was confirmed by Sanger sequencing.

Sanger sequencing. Exon 10 of the SLC26A4 gene was amplified from DNA samples of patients using primers 5ʹ-GAGCAGATATAGCATTTGATGATGAGATG-3ʹ and 5ʹ-CTCGGTGCGAGCCTTCCT-3ʹ. PCR products were purified with Agencourt Ampure XP magnetic beads, followed by cycle sequencing with the BigDye Terminator v3.1 Cycle Sequencing Kit (Applied Biosystems, USA). Sanger reaction products were purified by gel filtration on Sephadex G-50 medium columns (GE Healthcare, USA) and analyzed on an ABI 3130XL Genetic Analyzer (Applied Biosystems, USA).

Screening of the novel c.1185_1196dup variant in the control sample. The c.1185_1196dup variant in exon 10 was screened in the control sample by amplifying the corresponding DNA fragment with primers 5ʹ-GACCTCTCT CAGATGGTATGGCGT-3ʹ and 5ʹ-CTCGGT GCG AGC C TTCCT-3ʹ. PCR products were resolved on a 4 % agarose gel, allowing discrimination between wild-type (395 bp) and heterozygous (395 and 407 bp) alleles.

All genetic engineering procedures were performed at the Cell Technologies Sector of the Institute of Cytology and Genetics, Siberian Branch of the Russian Academy of Sciences (Novosibirsk). Sanger sequencing and MLPA analyses were carried out at the SB RAS Genomics Core Facility (D.G. Knorre Institute of Chemical Biology and Fundamental Medicine, Siberian Branch of the Russian Academy of Sciences, Novosibirsk).

**Bioinformatics analysis**

The functional impact of the novel c.1185_1196dup variant on the structure and function of the pendrin protein was predicted using the following bioinformatics tools: MutationTaster2021 (https://www.genecascade.org/MutationTaster2021/) (Steinhaus et al., 2021), PROVEAN (http://provean.jcvi.org) (Choi Y. et al., 2012, 2015), SIFT (https://siftdna.org/www/ SIFT_indels2.html) (Hu J. et al., 2013), VEST-Indel / CRAVAT (https://cravat.us/CRAVAT/) (Douville et al., 2015), and MutPred-Indel (https://mutpred2.mutdb.org/mutpredindel/) (Pagel et al., 2019). The secondary structure of pendrin was modeled using the I-TASSER platform (https://aideepmed. com/I-TASSER/) (Zheng et al., 2021, 2026; Zhou et al., 2022). Structural visualization of the pendrin protein was performed with DeepView / Swiss-PdbViewer v4.1.0 (http://www.expasy. org/spdbv/) (Guex and Peitsch, 1997).

## Results

MLPA analysis targeting the SLC26A4 gene was performed in a group of 13 Tuvinian patients previously classified as M1 (i. e., carrying a single recessive pathogenic SLC26A4 variant). MLPA enables the detection of copy number alterations, including deletions and duplications, in specific genomic regions. In three of 13 M1 patients, a similar copy number alteration was observed in the region of exon 10: the normalized ratio for probe 09251-L09442 (SALSA MLPA Probemix P280-B4 SLC26A4 kit) was approximately 0.5 (Fig. 1a). To further characterize the DNA samples from these three patients, fragments of exon 10 were subjected to molecular cloning into the pUC18 plasmid vector, followed by Sanger sequencing. Sequence analysis of the cloned fragments revealed a 12-nucleotide duplication (AGGATTCTTCTC) on one allele, corresponding to the c.1185_1196dup variant (Fig. 1b).

**Fig. 1.**
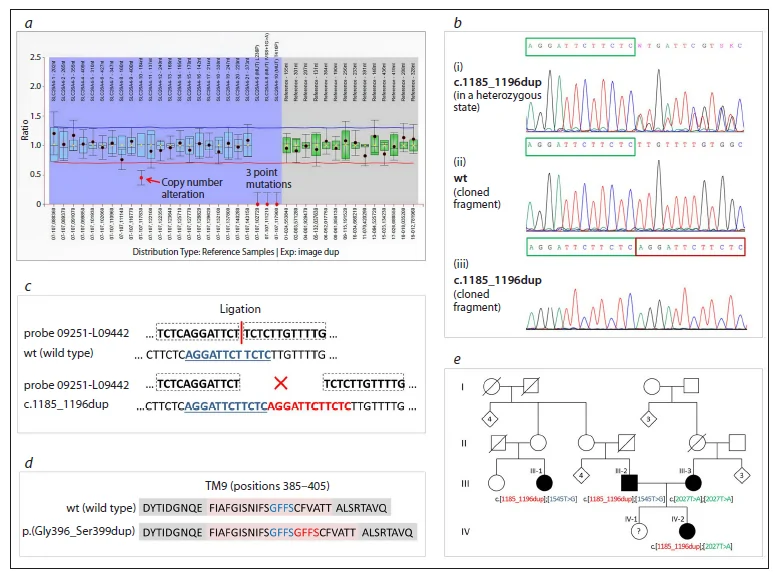
Identification and molecular genetic analysis of a novel variant c.1185_1196dup in the SLC26A4 gene. а – MLPA profile of one of the DNA samples with a copy number alteration in the region of exon 10 (shown by the red arrow) (see explanation in the text); b – Sanger sequencing traces revealing the c.1185_1196dup variant: (i) directly sequenced DNA fragment with the heterozygous c.1185_1196dup variant in exon 10; (ii) cloned wild-type (wt) fragment of exon 10; (iii) cloned fragment of exon 10 carrying the c.1185_1196dup variant; c – sequence fragments of probe 09251-L09442 (the SLC26A4 exon 10 region) from the SALSA MLPA Probemix P280-B4 SLC26A4 kit and the target region in the SLC26A4 gene: the wild-type (wt) is highlighted in blue, the 12-nucleotide insertion is shown in red (see explanation in the text); d – amino acid sequence of the pendrin protein region including transmembrane domain 9 (TM9); GFFS – four amino acid residues Gly-Phe-Phe-Ser: the wild-type sequence (wt) is shown in blue, the duplicated sequence resulting from c.1185_1196dup is highlighted in red; e – pedigree of Tuvinian families with individuals III-1, III-2, and IV-2 having compound heterozygous SLC26A4 genotypes. Variant c.1185_1196dup (exon 10) is highlighted in red, variant c.1545T>G (exon 14) – in blue, variant c.2027T>A (exon 17) – in green. Black symbols indicate individuals with hearing loss. “?” denotes unknown hearing status (individual IV-1).

The observed reduction in the MLPA signal (~0.5) for the 09251-L09442 probe in samples carrying the c.1185_1196dup variant is consistent with a heterozygous deletion (Fig. 1a). The 09251-L09442 probe (184 bp) contains two oligonucleotides (5ʹ-TCTCAGGATTCT-3ʹ and 5ʹ-TCTCTTGTTTTG-3ʹ) that hybridize adjacently to the target sequence in SLC26A4 (TCTCAGGATTCT-TCTCTTGTTTTG), enabling their subsequent ligation (Fig. 1c). Notably, the duplicated sequence (AGGATTCTTCTC) in the c.1185_1196dup variant overlaps with the probe sequence (Fig. 1c). In the heterozygous state of the c.1185_1196dup variant, the probe signal is detected only from the wild-type (wt) allele. This is because the 12-nucleotide duplication increases the distance between the two probe segments, thereby preventing their ligation and subsequent amplification. Consequently, the absence of a signal from the mutant allele results in an apparent reduction of the target copy number to ~0.5, masking the true nature of the variant as a duplication (Fig. 1a). This observation exemplifies a welldocumented limitation of the MLPA method: genetic variants that overlap with probe binding sites can interfere with hybridization and/or ligation, leading to false-positive fluorescent signals and potentially erroneous interpretation of the results. In such cases, it is recommended to continue research using other methods (Schouten et al., 2002; Stuppia et al., 2012; Varga et al., 2012). In the present study, MLPA initially identified three DNA samples with a copy number alteration (~0.5) in exon 10 of SLC26A4. Subsequent detailed characterization by molecular cloning and Sanger sequencing revealed that this alteration represented a heterozygous 12-nucleotide duplication (c.1185_1196dup). Further analysis demonstrated that c.1185_1196dup is an in-frame insertion in exon 10, resulting in the incorporation of four additional amino acid residues (Gly-Phe-Phe-Ser) within the ninth transmembrane domain (TM9, amino acid positions 385–405, UniProt: https://www. uniprot.org/uniprotkb/O43511/entry) of the pendrin protein (p.(Gly396_Ser399dup)) (Fig. 1d).

Three patients from two related Tuvinian families, who had previously been classified as monoallelic carriers (M1) for SLC26A4, were reclassified following the identification of the c.1185_1196dup variant. Their refined genotypes are as follows: III-1 – c.[1185_1196dup];[1545T>G], III-2 – c.[1185_1196dup];[1545T>G], and IV-2 – c.[1185_1196dup]; [2027T>A] (Fig. 1e). Notably, pedigree analysis (Fig. 1e) revealed the co-segregation of three distinct pathogenic SLC26A4 alleles: the novel c.1185_1196dup variant (exon 10); the unique c.1545T>G variant (exon 14), previously reported only in Tuvinian individuals; and the c.2027T>A variant (exon 17), which is relatively frequent in Tuvinians but rare elsewhere (Danilchenko et al., 2021).

Screening for the c.1185_1196dup variant in a control sample of 129 Tuvinian individuals without hearing impairment did not detect this variant (data not shown).

To evaluate the potential functional consequences of the c.1185_1196dup (p.(Gly396_Ser399dup)) variant on the structure and function of the pendrin protein, several bioinformatics tools were applied (see the Table). They were chosen by the need to apply algorithms specifically adapted for indel (insertions/deletions) prediction, as most available tools are primarily optimized for single nucleotide variants (SNVs). The majority of the applied prediction algorithms indicated a damaging effect of the c.1185_1196dup variant on the structure and function of pendrin (see the Table).

**Table 1.**
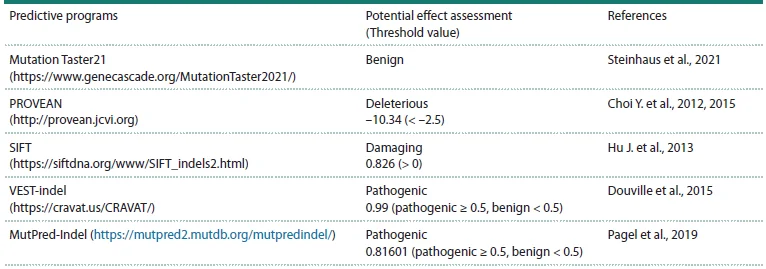
Bioinformatic assessment of the functional effect of the novel c.1185_1196dup variant in the SLC26A4 gene

## Discussion

In the present study, MLPA analysis of three M1 patients from two related Tuvinian families led to the identification of a novel variant in the SLC26A4 gene: NM_000441.2:c.1185_1196dup. This variant represents a 12-nucleotide tandem duplication in exon 10, classified as an in-frame insertion. It results in the incorporation of four additional amino acid residues (Gly-Phe- Phe-Ser) in transmembrane domain 9 (TM9) of the pendrin protein, designated as p.(Gly396_Ser399dup) (Fig. 1 and 2). Variant c.1185_1196dup was not detected in the control sample of Tuvinians (n = 129), and was also not registered in any of the world genomic databases (dbSNP, gnomAD, ClinVar).

**Fig. 2.**
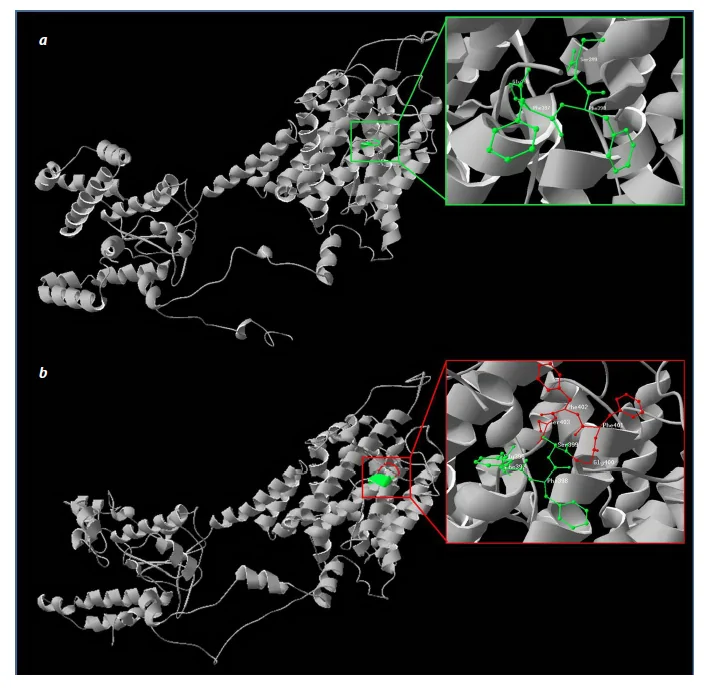
Schematic representation of the pendrin protein secondary structure and the fragments of its amino acid sequence. а – wild-type amino acid sequence 396Gly-397Phe-398Phe-399Ser (shown in green); b – duplicated sequence Gly396_Ser399dup (shown in red).

Currently, the total number of all identified variations in the SLC26A4 gene sequence (21 exons), spanning approximately 57 kb of genomic DNA (GRCh38, NC_000007.14, 7:107,660,828-107,717,809), exceeds 37,000 (the Deafness Variation Database: https://deafnessvariationdatabase.org/ genes/SLC26A4, May 2026). However, only approximately 6–7 % (~2,500 variants) have been classified according to current guidelines (Richards et al., 2015) as “pathogenic”, “likely pathogenic”, “benign”, or “likely benign”; the pathogenicity status of the vast majority remains uncertain. More than 800 variants with established pathogenicity (“pathogenic” and “likely pathogenic”) are linked to a broad phenotypic spectrum of hearing impairments. Notably, small insertions and deletions (one to several nucleotides) constitute a substantial proportion (~25 %) of pathogenic variants in the coding regions of SLC26A4. Most of these are frameshift variants that introduce premature stop codons and lead to truncated, nonfunctional proteins. In contrast, in-frame indels are considerably rarer and have been reported only in isolated cases of hearing loss (the Deafness Variation Database: https:// deafnessvariationdatabase.org/genes/SLC26A4, May 2026). These variants do not disrupt the reading frame but alter the length of the pendrin protein.

The precise molecular structure of human pendrin, a member of the SLC26 family of anion transporters (SLC26A1–11), has not yet been determined. However, structural insights into human pendrin have been advanced by cryo-electron microscopy (cryo-EM) studies of orthologous proteins in several mammalian species (Mus musculus, Sus scrofa), which show significant conservation with human pendrin (Walter et al., 2019; Liu et al., 2023; Takahashi, Homma, 2024; Wang et al., 2024). Current structural models indicate that human pendrin functions as a homodimer, with each protomer comprising 14 transmembrane α-helices (TM1–TM14) and a C- terminal cytoplasmic STAS domain (Sulfate Transporter and Anti- Sigma factor antagonist domain), whose precise function remains incompletely understood. The transmembrane region adopts an inverted repeat structure formed by TM1–TM7 and TM8–TM14. The core domain (TM1–TM4 and TM8–TM11) harbors two anion-binding sites and is directly responsible for substrate transport, whereas a smaller scaffold (or gate) domain (TM5–TM7 and TM12–TM14) provides structural support and functional coordination for the core domain (Liu et al., 2023; Wang et al., 2024).

The p.(Gly396_Ser399dup) variant resulting from c.1185_1196dup introduces an insertion within TM9, a transmembrane segment located in a central position (385–405) in the pendrin protein (780 amino acid residues). TM9 and its adjacent segments are highly conserved regions of the protein (Fig. 3), as evidenced by the high density of pathogenic and likely pathogenic SLC26A4 variants in these regions (the Deafness Variation Database: https://deafnessvariationdatabase.org/ genes/SLC26A4, May 2026).

**Fig. 3.**
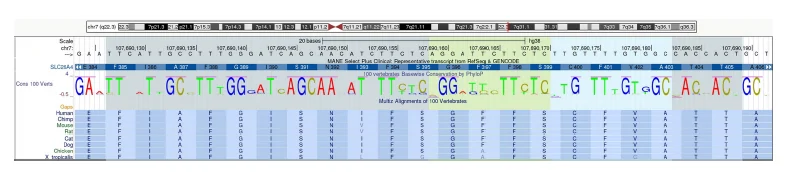
Multiple sequence alignment of the pendrin protein generated using the UCSC Genome Browser (https://genome.ucsc.edu/). The transmembrane segment TM9 (amino acid positions 385–405, according to UniProt: https://www.uniprot.org/uniprotkb/O43511/entry) is shaded in gray. The wild-type nucleotide and amino acid sequences are shown in green; the insertion site corresponding to the c.1185_1196dup variant is highlighted in blue.

Thus, the “shift” of the amino acid sequence in the case of the p.(Gly396_Ser399dup) variant, which occurs in the transmembrane segment TM9 and leads to an increase in the length of the pendrin protein from 780 to 784 amino acid residues, can probably change the secondary structure of the protein, which entails a disruption or complete loss of the anion transport function.

Unlike frameshift indels, which typically result in premature translation termination and loss of protein function, in-frame indels alter the length of a protein while leaving the underlying amino acid sequence intact. The larger the size of such indels and the more evolutionarily conserved the amino acid residues within the indel, the more significant their negative effect on protein structure and function may be. The functional impact of such variants is often challenging to predict, because most bioinformatics tools are optimized for missense substitutions rather than indels, although algorithms for in-frame indel prediction are constantly being improved (Pagel et al., 2019; Cannon et al., 2023; Abderrazzaq et al., 2026). According to established variant interpretation guidelines (Richards et al., 2015), in-frame indels located in non-repetitive, evolutionarily conserved regions and resulting in the synthesis of a protein of altered length, may be assigned a moderate pathogenicity criterion (PM4). Most of the predictive programs used to evaluate the novel variant c.1185_1196dup (p.(Gly396_Ser399dup)) predict its damaging effect on the structure and function of the pendrin protein (see the Table). The pathogenicity of this variant is also supported by its segregation with hearing loss in three Tuvinian patients, in whom it was found in a compound heterozygous state with other known pathogenic variants of the SLC26A4 gene (Fig. 1e), as well as its absence in world genomic databases (dbSNP, gnomAD, ClinVar). A limitation of the present study is the absence of functional in vitro assays to directly assess the impact of p.(Gly396_Ser399dup) on the structure and functions of protein pendrin. In the future, we plan to conduct such studies based on experimental platforms designed taking into account the transmembrane localization of the pendrin protein and its functioning as an anion transporter.

## Conclusion

The molecular diagnosis of patients harboring a single identified pathogenic recessive allele of the SLC26A4 gene (M1 patients) remains a significant challenge, as conventional molecular genetic approaches may fail to detect all underlying variants. In this study, reanalysis of the SLC26A4 locus by MLPA in a cohort of 13 M1 patients enabled the identification of a novel variant, c.1185_1196dup, in three individuals. The c.1185_1196dup variant represents an in-frame 12-nucleotide insertion resulting in the incorporation of four additional amino acid residues Gly-Phe-Phe-Ser (p.(Gly396_Ser399dup)) within a highly conserved transmembrane domain (TM9) of the pendrin protein. The combined data on the novel c.1185_1196dup variant, including its segregation with hearing loss, consistent prognostic estimates obtained by various bioinformatics tools, and its absence in global databases, support its probable pathogenicity. However, functional in vitro studies are needed to confirm the potential negative effects of novel c.1185_1196dup (p.(Gly396_Ser399dup)) variant.

## Conflict of interest

The authors declare no conflict of interest.
